# Consensus statement—graft treatment in cardiovascular bypass graft surgery

**DOI:** 10.3389/fcvm.2024.1285685

**Published:** 2024-02-27

**Authors:** Maximilian Y. Emmert, Johannes Bonatti, Etem Caliskan, Mario Gaudino, Martin Grabenwöger, Martin T. Grapow, Paul Phillip Heinisch, Teresa Kieser-Prieur, Ki-Bong Kim, Attila Kiss, Fatima Mouriquhe, Markus Mach, Adrianna Margariti, John Pepper, Louis P. Perrault, Bruno K. Podesser, John Puskas, David P. Taggart, Om P. Yadava, Bernhard Winkler

**Affiliations:** ^1^ Department of Cardiothoracic and Vascular Surgery, Deutsches Herzzentrum der Charite (DHZC), Berlin, Germany; ^2^ Institute for Regenerative Medicine (IREM), University of Zurich, Zurich, Switzerland; ^3^ Department of Cardiothoracic Surgery, UPMC Heart and Vascular Institute, University of Pittsburgh, Pittsburgh, PA, United States; ^4^ Department of Cardiothoracic Surgery, Weill Cornell Medicine, New York, NY, United States; ^5^ Sigmund Freud Private University, Vienna, Austria; ^6^ Department of Cardiovascular Surgery KFL, Vienna Health Network, Vienna, Austria; ^7^ Hirslanden Klinik Zürich, HerzZentrum, Zürich, Switzerland; ^8^ German Heart Center Munich, Technical University of Munich, School of Medicine, Munich, Germany; ^9^ LIBIN Cardiovascular Institute of Alberta, University of Calgary, Calgary, AB, Canada; ^10^ Department of Thoracic and Cardiovascular Surgery, Seoul National University Hospital, Seoul National University College of Medicine, Seoul, Republic of Korea; ^11^ Ludwig Boltzmann Institute at the Center for Biomedical Research, Medical University of Vienna, Vienna, Austria; ^12^ Montreal Heart Institute, Montreal, QC, Canada; ^13^ Department of Cardiac Surgery, Medical University Vienna, Vienna, Austria; ^14^ The Wellcome-Wolfson Institute of Experimental Medicine, Belfast, United Kingdom; ^15^ Cardiology and Aortic Centre, Royal Brompton Hospital, Royal Brompton and Harefield NHS Foundation Trust, London, United Kingdom; ^16^ Department of Cardiovascular Surgery, Mount Sinai Morningside, New York, NY, United States; ^17^ Nuffield Dept Surgical Sciences, Oxford University, Oxford, United Kingdom; ^18^ National Heart Institute, New Delhi, India; ^19^ Karld Landsteiner Institute for Cardiovascular Research Clinic Floridsdorf, Vienna, Austria

**Keywords:** CABG, endothelium, heart, radial artery, VEST

## Abstract

Coronary artery bypass grafting (CABG) is and continues to be the preferred revascularization strategy in patients with multivessel disease. Graft selection has been shown to influence the outcomes following CABG. During the last almost 60 years saphenous vein grafts (SVG) together with the internal mammary artery have become the standard of care for patients undergoing CABG surgery. While there is little doubt about the benefits, the patency rates are constantly under debate. Despite its acknowledged limitations in terms of long-term patency due to intimal hyperplasia, the saphenous vein is still the most often used graft. Although reendothelialization occurs early postoperatively, the process of intimal hyperplasia remains irreversible. This is due in part to the persistence of high shear forces, the chronic localized inflammatory response, and the partial dysfunctionality of the regenerated endothelium. “No-Touch” harvesting techniques, specific storage solutions, pressure controlled graft flushing and external stenting are important and established methods aiming to overcome the process of intimal hyperplasia at different time levels. Still despite the known evidence these methods are not standard everywhere. The use of arterial grafts is another strategy to address the inferior SVG patency rates and to perform CABG with total arterial revascularization. Composite grafting, pharmacological agents as well as latest minimal invasive techniques aim in the same direction. To give guide and set standards all graft related topics for CABG are presented in this expert opinion document on graft treatment.

## 1 Introduction

**Figure 1:**
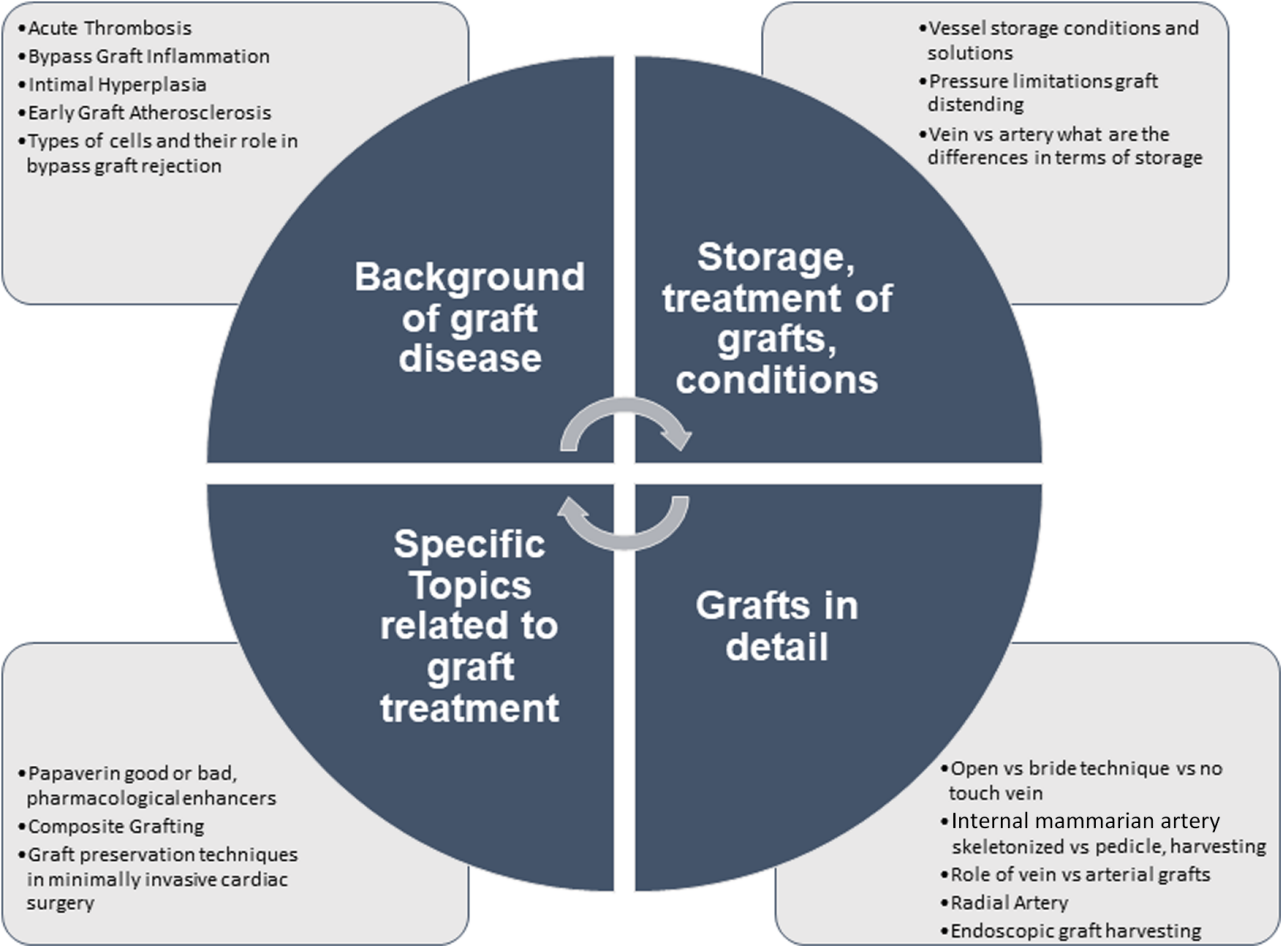
Overview graft treatment, handling and related topics.

Just as coronary bypass surgery, with its associated improved postoperative quality of life and life expectancy, represents a pivotal positive milestone in the life of a patient with significant coronary artery disease, the development of bypass graft failure constitutes a detrimental negative landmark in the life of patients after coronary bypass surgery (1) (Figure 1).

Unlike the left internal thoracic artery (LITA), which has been shown to have an excellent long-term patency rate but is limited by its length and therefore often not suitable for multiple grafts, the saphenous vein graft (SVG) is available in longer lengths and allows revascularization of additional coronary arterial vessel territories. For this reason, the SVG still remains the most commonly used conduit for CABG, although a multi arterial or even complete arterial revascularization approach is being advocated lately (2, 3). The most frequently used conduits for this purpose are the radial artery and the right internal mammary artery (1).

The transition to a complete-arterial revascularization approach was initiated primarily by the higher rate of short-term graft failure of SVGs as well as a lower long-term patency rate, which led to lower event-free survival and long-term survival and compared with arterial grafts (4, 5). As a consequence, there has been a legitimate interest in a deeper understanding of the underlying pathophysiology in the development of vein graft disease, and various preventive therapies have been developed over the years, only few have been successful enough to be adopted on a widespread basis. Much of this is due to our limited understanding of the complex pathological interplay involved in the development of the disease. As possible components of this process, broad thematic complexes such as vascular inflammation, intercellular signaling pathways in both venous and arterial bypass conduits, superimposed atherosclerosis, and the influence of numerous circulating cells and molecules are currently being discussed. This overall complex process of various interactions between multiple factors, attributable to either the patient or the surgical technique, affecting the pathophysiology as well as the clinical impact of this disease, makes the study of these contributing factors particularly challenging (1, 6–8).

## 2 Background of graft disease

**Figure 2:**
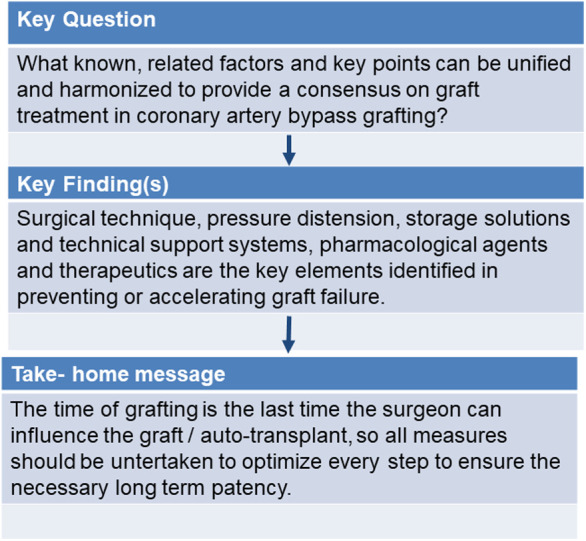
Visual abstract graft treatment in coronary artery bypass surgery.

Four interrelated pathways form the basic pathophysiological framework of bypass graft failure: acute bypass graft thrombosis, intimal hyperplasia, graft inflammation, as well as early graft atherosclerosis (6, 8) (Figure 2).

### 2.1 Acute thrombosis

Acute thrombosis is responsible for the majority of early vein graft failures (7, 8). Surgical injury during the conduit harvest resulting in wall distention, occurrence of high shear stress and damage to the vasa vasorum with consecutive hypoxia can lead to endothelial dysfunction and activation, resulting in an intravascular prothrombotic state whereby the de-endothelialized vessel's extracellular matrix (ECM) is exposed to luminal blood components (6, 9). This leads to increased expression of adhesion molecules and vasoconstriction via a marked reduction in prostacyclin (PGI2), nitric oxide (NO), thrombomodulin, and heparin-like substances in the injured endothelial cells (EC) (6, 9). The coagulation cascade is activated, resulting in platelet activation and adhesion to de-endothelialized areas, producing various pro-coagulation factors such as thromboxane A2, fibronectin, fibrinogen, thrombospondin, platelet factor IV, von Willebrand factor, and b-thromboglobulin Harskamp (6, 8, 10–12). Although reversible to a certain degree, these processes can lead collectively to 3%–12% early graft occlusion (8, 11, 13).

### 2.2 Bypass graft inflammation

At any given postoperative stage, some degree of concomitant localized acute or chronic inflammation may occur, aggravating any pathophysiologic progression of graft failure.

In the initial phase of this process, the extracellular matrix exposed to the vessel lumen by de-endothelialization binds leukocytes and platelets that infiltrate the intima (11). Consecutive or concomitant intimal hyperplasia is enhanced by growth factors and released cytokines (IL-1, IL-6, and TNF-alpha). Subsequently, monocytes infiltrate the IH layer, differentiate into macrophages, and ultimately develop into foam cells, as they would in atherosclerosis due to lipid uptake. The localized chronic inflammatory changes allow this process to occur more rapidly than in native arteries. Matrix metalloproteinases secreted by intimal macrophages lead to cleavage of the extracellular matrix (ECM), which in turn leads to migration of vascular smooth muscle cells (VSMC) into the intima and further induces their proliferation, triggers further inflammation and accelerates atherosclerosis (14).

Furthermore, damage to the perivascular lipid layer during graft harvesting results in the release of cytokines, which in turn recruit lymphoid cells to the adventitia of the bypass graft, which in turn initiate a proinflammatory process in the vessel by further cytokine secretion. In addition, destruction of the perivascular fat leads to reduced bioavailability of NO, which has a regulatory effect on the vascular tone (11, 14).

Although re-endothelialization occurs early postoperatively, the process of intimal hyperplasia remains irreversible. This is due in part to the persistence of high shear forces, the chronic localized inflammatory response, and the partial dysfunctionality of the regenerated endothelium (7, 12, 14).

### 2.3 Intimal hyperplasia

Vascular tone, cell proliferation, and response to inflammation are regulated by the constant interaction between ECs and VSMCs, where in a healthy state this interaction keeps VSMCs in a quiescent state (15). Intimal hyperplasia as a chronic disease, is primarily characterized by the transition of VSMCs in the medial layer from a resting state to a synthetic proliferative state, which consequently leads to a hyperplasia of the intimal layer (9, 16). This transition can be exacerbated by local inflammation and endothelial injury, as this causes cytokine and interleukin releaser and ECs to enter a prothrombotic state, further disrupting this interaction (11, 14).

While increased ECM protein secretion by ECs and VSMCs as indirect communication pathway expedite VSMC phenotype switching, this process can also be induced via EC-derived micro RNAs (miR-126), p38 mitogen-activated protein kinase (p38) and Nuclear Factor Kappa Light Chain Enhancer of Activated B Cells (15, 17–20). In addition, Kruppel-like factors 4 and 5, among others, have been shown to have a significant effect on IH development. KLF4 can induce downregulation of contractile markers of VSCMs, thereby converting these cells to a synthetic state, but can induce upregulation of anti-inflammatory and antithrombotic factors when overexpressed in endothelial cells. KLF-5 is expressed in response to vascular injury in de-differentiated VSMCs and is also found to lead to proliferation activation (21). On the other hand, endothelium-derived NO helps regulate the tone of the medial layer and suppresses the phenotypic conversion of VSMCs to a proliferative state (15).

The complex development process of intimal hyperplasia caused by the interaction of ECs and VSMCs has not yet been fully elucidated. Thus, through extensive research in this area, it is anticipated that new and sustainable therapeutic approaches aimed at reducing postoperative bypass graft failure can be developed.

### 2.4 Early graft atherosclerosis

Atherosclerotic disease, which progresses much more rapidly in bypass grafts compared with native arteries, differs primarily in accelerated lipid uptake as well as slower lipolysis (22).

In addition, atherosclerotic plaques in bypass grafts are composed of increased numbers of foam cells and other inflammatory cells (e.g., multinucleated giant cells). In venous bypass grafts, some of these cells have been shown to originate from venous VSMCs rather than circulating cells, as had been assumed. The associated intimal hyperplasia thus becomes an important cofactor for the progression and severity of atherosclerosis in venous grafts (22).

In contrast to native artery atherosclerosis, vein graft atherosclerosis tends to be diffuse, concentric, with little or no calcification and fragile fibrous cap, making it more susceptible to plaque-rupture (6, 14, 23, 24).

#### 2.4.1 Types of cells and their role in bypass graft failure

Studying the types of cells involved in bypass grafts can also help with understanding the mechanisms surrounding bypass graft failure and its treatment or prevention.

There are two main categories of cells involved in bypass graft failure, which may be acute or chronic, the latter being the principal cause of graft pathologies:

(a) Specific cells such as T cells and B cells, which drive the antigen-based reaction;
(b) Non-specific cells such as ECs, platelets, NK cells, VSMCs, macrophages, foam cells and other inflammatory cells.

In the first category, T cells and B cells, which greatly contribute to the acute chronological phase of graft pathologies, can act either in combination or independently. The T cell response directly leads to acute cell graft pathologies while the B cell process leads to acute humoral ractions (25). In addition, B cells interact with T cells on transplanted tissues and organs through antigen presentation and cytokines generation (26). Dendritic Cells (DCs), which can initiate naïve T cells, are also involved in the antigen-specific graft failure process; they are activated by signals eluding to danger, which are mediated by pattern-recognition receptors (PRRs) (25). Furthermore, DC-EC interactions further complicate the immunogenic process initiated by ECs (26).

In the second category of cells involved in graft failure, a multiphase process takes place, which is also involved in the progression and development of atherosclerotic disease. This process is characterized by EC activation and dysfunction, platelet activation and aggregation, lipid accumulation, expression of adhesion molecules and monocyte infiltration into the vessel wall followed by conversion to lesional macrophages and foam cells (27). In the case of NK cells, which are central innate immune system components, early activation post graft transplantation may lead to targeting and destruction of allogenic cells. NK cells are well known viral infection and tumour suppressors but they can also recognize transplanted grafts and differentiate from self and non-self backgrounds despite not expressing germline-encoded antigen receptors (26). Their role in graft failure varies based on the type of allograft involved with recent studies reassessing the previously held belief of NK cells being of negligible risk. Furthermore, VSMCs, which play a crucial role in the function of the cardiovascular systems, when under the influence of atherogenic stimuli, phenotypically switch from a non-proliferative profile, typically seen in non-diseased arteries, to a proliferative phenotype with the ability to migrate and affect ECM production leading to vessel remodeling (26). Moreover, it's important to highlight the VSMCs are vital parts in vessel tissue engineering. For example, embryonic lung fibroblasts gave rise to VSMCs after direct reprogramming through DKK3 signaling; using this method in the adventitia and lumina, VSMCs layered the newly created vessel walls together with surface ECs. In a similar approach human fibroblasts were reprogrammed towards ECs (28).

## 3 Storage, treatment of grafts, conditions

### 3.1 Vessel storage conditions and solutions

During the last almost 60 years SVG together with the IMA have become the standard of care for patients undergoing CABG surgery (29). While there is little doubt about the benefit for our patients, the patency rates are constantly under debate. Arterial grafts show a significantly higher patency (up to 90% at ten years), the use of both IMAs seems to be superior but this has to be paid by a higher risk of initial sternal infections (30). Whether total arterial revascularization further improves quality of life and survival still has to be proven in large, multicenter trials. Although SVGs have a proven patency of only around 60% after 10 years, they remain the most important and most widely used conduit in CABG surgery worldwide. Consequently, there is considerable interest to look into the pathophysiology of graft failure, thereby developing new strategies to protect the SVG (4). Little has changed since the first veins were harvested and stored during the early 1960s. Only during the 1980s the first studies proved that flushing with saline, high distension pressures and the effects of different handling techniques induced severe and deleterious injury of the endothelium. The authors concluded that techniques with minimal manipulation during harvesting, immersion in 4°C cold heparinized autologous blood and a distension pressure of less than 100 mmHg resulted in the best preserved endothelium (31). However, also storage in autologous blood has shown controversial results (32). Therefore, it is no wonder that there is increasing interest for alternative storage and protection solutions with or without pharmacological adjuncts (33). Promising studies used the University of Wisconsin solution (UWS), originally designed as intracellular storage and harvesting solution for transplantation. Veins stored in UWS showed functional endothelium as compared to controls (34). As an interesting solution, TiProTec was proposed. Only for ex vivo use, veins treated with this storage solution showed favorable preservation of NO-dependent relaxation up to 24 h after harvest but up to now no clinical use has been reported and the use was surprisingly suspended by 2023 by the supplier after a first in human study was conducted (35). A subgroup analysis of the PREVENT IV randomized trial demonstrated that buffered saline solution had lower vein graft failure rates when studied by angiography 12–18 months after surgery and better clinical outcomes compared to physiological saline or autologous blood solutions (36). The surgical factor e.g., brisk handling, overdistension or strechting of the grafts was the strongest factor associated with vein graft failure in this study. More recently, adding antioxidants (glutathione, L-ascorbic acid) as well as substrates for NO-synthesis (L-arginine) to a standard physiologic salt solution, this so-called GALA solution was used in a large study in patients undergoing CABG in the Veterans Affairs Healthcare System in Boston (37). The results were promising and led to the introduction of a follow-up solution based on GALA, commercialized as DuraGraft in 2014. In the meatime more evidence has become available that DuraGraft can prevent intima hyperplasia at 1 year using multidetector computed tomography (38). Finally, first results from ex vivo studies on preservation of radial artery grafts are available and point into the same direction as well as large data sets from a multicenter registry (39). Currently Duragraft is the only available specific storage and treatment solution.

### 3.2 Pressure limitations graft distending

The suboptimal long-term patency rate of autologous human saphenous vein grafts use in CABG surgery is mainly due to intimal hyperplasia (IH) and atherosclerosis leading to vessel lumen narrowing, graft stenosis and occlusion (40–43). These pathological processes ultimately cause venous graft failure (VGF) both in the short term (30 days to 2 years) and long term (>2 years), respectively (42). Venous wall adaptation to a greater intraluminal pressure of arterial blood flow and endothelial trauma incurred to SV conduits during back-table graft preparation, namely storage in acidic or non buffered preservation solutions, usage of surgical skin markers and pressure distension when flushing the vein with uncontrolled pressure by handheld syringes, are among the foremost contributors to endothelial denudation that potentiate inflammatory responses leading to IH (41, 42, 44–49). Pressure distention has been part of the standard graft preparation procedure for decades since it increases luminal diameter, helps identify leaks or injuries, prevents spasm, and facilitates anastomosis (15, 42, 50). This procedure is still used despite the risk of damage to the medial smooth muscle layer responsible for smooth muscle cells apoptosis and dedifferentiation in addition to upregulating the expression of endothelial adhesion molecules (ICAM-1, VCAM-1, and P-selectin) which occur following a distension pressure of 300 mmHg in HSV, contributing to early VGF (44, 51–53). When comparing unmanipulated and manipulated HSV grafts used in CABG surgery, RT-PCR and immunohistochemical imaging both reveal the same findings: increased expression of several innate cell markers of inflammation such as toll-like receptors (TLRs) and scavengers' receptors on macrophages responsible for the uptake of modified low-density lipoproteins (LDLs) promoting progression of atherosclerotic lesions (41). Vascular reactivity studies using organ chamber apparatus allow quantification of smooth muscle relaxation by force transducers following exposure of submerged SV rings to agonists such as acetylcholine or other cholinomimetics, leading to NO generation by eNOS which initiates c-GMP-mediated vascular smooth muscle relaxation. In a porcine model used to compare vascular reactivity in unregulated distended (pressure in excess of 600 mmHg) SV vs. in-line pressure release valve (PRV) SV (maximum pressure of 140 mmHg) (54), distended SV were functionally impaired, while function was preserved in SV grafts in which distension pressure was regulated, suggesting that PRV preserves the endothelial monolayer (44, 54). In 2014, Li et al. observed decreased physiological contraction responses to phenylephrine, as well as decreased endothelial-dependent physiological relaxation in HSV distended with a hand-held syringe in comparison with HSV distended with PRV and non-distended control tissue (44). Cheung-Flynn et al. reported that decreased vasomotor function after standard back table preparation of HSV was associated with significant alterations in multiple metabolic pathways, particularly those reflective of oxidative stress, phospholipid hydrolysis, and energy depletion (55). In the same study, the use of an optimized preparation (OP) technique consisting of pressure distending with PRV, marking with non-toxic water soluble P2X7R antagonist brilliant blue FCF, and storing in a balanced buffered electrolyte solution mitigated the deleterious changes triggered by standard vein preparation (55, 56). Taken together, surgeons should consider no distension of SV grafts for optimal results. If distension is preferred this should be done with pressure controlled apparatus to minimize graft wall trauma and its ensuing potential complications.

### 3.3 Vein vs. artery what are the differences in terms of storage

Despite its acknowledged limitations in terms of long-term patency, the saphenous vein is currently the most often used graft in CABG as it possesses unique natural features and certain intrinsic degeneration, which can have an effect on its eventual performance (33, 57).Vein grafts are continually adapting conduits that generate intimal hyperplasia in response to the arterial circulation. Nowadays, the primary limitation to more durable grafts is intimal hyperplasia. *In vitro* evidence indicates that intraoperative preservation solutions may have an effect on endothelial function. There is inconsistent evidence about the usage of saline or blood-based products in the available current research, necessitating equivalent large randomized trials. An in-depth analysis of the current literature concluded that the University of Wisconsin solution, for example, may be favorable when compared to both blood and crystalloid solutions. While saline solutions were proven to be detrimental to the endothelium, autologous whole blood exhibited some drawbacks (58). Once removed from the circulatory system, (autologous whole blood) AWB was observed to decrease its protective qualities. There are essentially three relevant reports that indicate no difference between saline and AWB. The first trial, conducted in mongrel dogs and included arterialization of the jugular veins, was unable to demonstrate a long-term benefit of AWB following arterialization. The combined the effects of continuous perfusion and endoscopic vein harvesting: the endoscopic method can have a significant effect on the endothelium (59). Only one study concluded that AWB has no effect on vascular reactivity and is inferior to saline (32). For the purpose of storing arterial conduits, heparinized autologous whole blood should be used that has bathed the endothelium of the arteries in its native anatomic position before removal. This might help to maintain both endothelial and contractile function in the conduit. It is recommended to avoid using normal saline solution because it has been demonstrated that it causes endothelial integrity to be disrupted, which in turn impairs functionality (60). Arterial grafts stored in heparinized autologous whole blood are found to be more sensitive to agonists than graft stored in saline solutions (60–62). The comparison of freshly harvested distal radial arterial grafts with surgically prepared proximal segments stored in heparinized whole blood containing papaverine until grafting, revealed a significant difference in contractile properties between the proximal and distal arterial segments. This suggests that the increase in contractility observed is due to the proximal segment's greater contractile properties, not storage in heparinized whole blood (62). The addition of vasodilators, such as papaverine, verapamil, or nitrates, to the storage solution, which is a common practice in all cardiac surgical units, may alter the effect of the storage solution on endothelial function (60). The fact that increased distension pressures or overdistension outweigh any benefit or disadvantage of whole blood or particular storage solutions over saline is critical. Saline exacerbates the effect of high distension pressures, particularly when at or above room temperature (33).

While novel or alternative solutions for storage demonstrate promising outcomes and were developed to address the aforementioned drawbacks, the literature on these products is still at a certain point elaborating and their clinical application is still underdeveloped (33). The custom-built individual storage solution appears to be the next obvious step, but massive obligatory trials will be difficult and expensive to conduct.

## 4 Grafts in detail

### 4.1 Open vs. bride technique vs. no touch vein

The open technique is the traditional and most commonly used technique for saphenous vein graft harvesting. It involves identification and exposure of the saphenous vein, routinely at the lower leg, by a small skin incision proximal to the medial malleolus (63). Stepwise small incisions continue along the course of the saphenous vein after dissection of subcutaneous tissue and adventitial layers including the thin facia above the saphenous vein. Ligatures (3-0 to 5-0 polyfilament sutures) or vascular titanium clips should be placed at a safe distance for side branches. The saphenous vein is completely freed from the underlying bed by blunt or sharp dissection until the desired length is obtained. Following systemic heparinization, the vein is divided and carefully cannulated with a metallic or plastic cannula at the distal end and finally divided at the proximal end. Gentle flushing without overdistension can be performed to check for untreated side branches or tears. Remaining leaky side branches are occluded with ligatures or vascular titanium clips and small tears are treated with 7-0/8-0 polypropylene sutures if necessary.

The bridging technique involves multiple small skin incisions instead of a long incision leaving intact bridges of skin under which the vein is dissected and freed from surrounding tissue by tunnels. This technique aims to facilitate better wound healing, lower the risk of bleeding, infection or other wound complications by an overall smaller wound area.

With the no-touch vein harvesting technique, the saphenous vein is harvested as a pedicle with the aim of preservation of vessel wall integrity by preserving the complete vasa vasorum and nerves in a continuous cushion of adventitia and perivascular adipose tissue (64). Endothelial damage with intimal loss and biochemical and functional changes caused during vein harvesting is thought to play an incremental role in the patency of saphenous vein grafts (65). Further, significant morbidity may be associated with the harvesting technique of saphenous vein grafts with increased wound healing complications not only in patients with diabetes and peripheral vascular disease. Several approaches have been suggested in the past to overcome these drawbacks. As such, endoscopic vein harvesting, the utilization of the bridging-technique and lately the no-touch vein harvesting technique have emerged as alternatives to the open harvesting technique. While the bridging technique reduces the wound area thus reducing postoperative wound complications, the evidence on graft patency is scarce. Khan et al. demonstrated in a comparative analysis of saphenous vein graft harvesting techniques reduced postoperative leg morbidity and increased patient satisfaction associated with the bridging technique, however, an analysis of graft patency and overall effect on mortality is missing (66).

The risk of endothelial damage during harvesting due to potentially increased mechanical trauma/extensive manipulation while preparation under the skin bridges is more likely with the bridge-technique as compared to the open vein harvesting and may thus not provide an overall advantage with regards to vein graft failure. The no-touch technique may provide here an interesting alternative. Since its introduction in 1996 by Souza, the no-touch preparation techniques has been investigated in numerous studies (67, 68). In a longitudinal randomized trial comparing the graft patency of conventionally harvested vein grafts with vein grafts harvested with the no-touch harvesting technique, Souza et al. demonstrated that the saphenous vein with surrounding tissue provides high short- and long-term patency rates comparable to the left internal mammary artery (LIMA) (69). Later, Samano and colleagues reported that the no-touch saphenous vein harvesting technique maintained a patency, after 16 years, superior to the conventional harvesting technique and still comparable to patency rates of the LIMA (70). However, a propensity-matched cohort analysis and a recent randomized controlled trial revealed no superiority for graft patency and clinical outcomes (71, 72).

Further, a major drawback of this technique, the higher incidence of wound leg complications, remains.

Despite more than a half of a century of CABG surgery and huge milestones taken to improve clinical outcomes, the issue of the right harvesting technique remains controversial. Large-scale randomized clinical trials and well-designed observational registries with long-term follow-up are warranted to elucidate more on the proper technique of vein graft harvesting.

### 4.2 Mammarian artery skeletonized vs. pedicle, harvesting

The internal mammary artery, (IMA) is a drug eluting conduit and the best substitute for diseased coronary arteries (73). Harvest techniques include the pedicle technique whereby the IMA is harvested with surrounding tissue, IM veins, intercostal muscle and parietal fascia, and the skeletonization technique in which only the IMA is removed from the chest wall. In an anatomical study of sternal blood supply, De Jesus noted that all 6 vessel types of the IMA are removed with the pedicle technique but three types, collateral in nature remain in place with skeletonization thereby anatomically explaining skeletonization's reduced rate of sternal infection (74). Three methods of skeletonization exist: use of the cautery tip as a dissector with or without low power, and the third uses the harmonic scalpel. Comparative studies of the pedicle vs. skeletonization techniques have shown that skeletonization reduces deep sternal infection (75–77), increases both free and post-anastomosis flow of the IMA and increases conduit length by 2–3 cm (78). Kieser et al. found harmonic harvest to reduce spasm, used fewer clips and was less damaging. Harmonic skeletonization is also the quickest technique: pedicled—19 min; cautery tip IMA skeletonization—32.2 min and harmonic skeletonization—15.4 min (10). Currently less than 10% of surgeons utilize harmonic technology (79–81). Also, the lateral thermal spread of electrocautery is 10 times that of the harmonic scalpel: electrocautery contact for 1 s damages 1 mm of tissue whereas harmonic scalpel contact for 1 s damages 0.1 mm of tissue. If surgeons in the COMPASS trial were using either cautery tip dissection without or without power, damage to IMAs with ensuing reduced graft patency might be more likely to occur (82). So far this is one of the most recent evidence for harvesting the internal mammary artery during CABG surgery using a skeletonised technique and speaks more in favor of the traditional pedicled technique but the surgical teams experience is an involved and importnat aspect in this regard (82). The IMA is the very best conduit; however, it is not indestructible and commands a respectful method of harvest.

### 4.3 Role of vein vs. arterial grafts

Saphenous vein grafts (SVG) remain the most commonly used conduits in CABG since their introduction to cardiac surgery (83). Their advantages compared to arterial grafts are the ease of use, abundance and their resistance to competitive flow. However, exposure of the SVG to the hemodynamics of the arterial circulation initiates a well-documented pathological cascade which limits SVG longevity. Contemporary studies show that 50% of SVG are occluded 10 years after CABG (84). With improved contemporary pharmacological therapy, the failure rates are reported in other studies better than 50% but still inferior to arterial grafts (85). SVG failure after CABG has two distinct phases with different etiologies. 10%–15% of SVG fail early due to technical errors, intrinsic poor quality or endothelial trauma during harvesting causing acute thrombosis. In contrast, long-term failure results from a high flow and pressure environment, that induces intimal hyperplasia, lumen irregularities and accelerated atherosclerosis (84). External SVG supports, a focus of research over the past 50 years, are intended to minimize these pathophysiological changes that adversely affect graft morphology and function culminating in long-term failure (86). Different stent devices, tested extensively in animal models, have confirmed their biomechanical benefits including reductions in SVG wall tension, dilatation and lumen irregularities leading to improved flow pattern (86). Subsequently, clinical studies led to both a substantial development in their design accompanied by the accumulation of a large body of evidence attesting to their benefit in CABG. Data from randomized controlled studies in CABG have consistently confirmed the biomechanical effects and benefits of external stenting on the progression of SVG disease. This includes improved Fitzgibbon perfect angiographic patency, a strong predictor of long-term patency, (odds ratio, 2.02; *P* = 0.03) and reduced intimal hyperplasia volume at 1-, 2- and 4.5-years post CABG by 15%-22% (*P* < 0.001) (87–89). Computational flow studies also confirmed the improved hemodynamics and optimal coherence tomography confirms improved lumen uniformity and reduced thrombus formation (90, 91). A recent observational study reported patency rates of external stents of 98% at 6–12 months with off pump CABG (92). While it is too early to determine the exact effect of external stenting on long term SVG patency and clinical outcome, intimal hyperplasia and SVG lumen irregularities are well validated pathologies which have critical roles in the development of SVG disease and have been shown to correlate adversely with long-term patency (93, 94). The safety profile of the latest generation external stents, and the fact that external stenting is the only technology to date that yielded consistent effectiveness in reduction of intimal hyperplasia and lumen irregularities up to 4.5 years after CABG, suggests that these technologies should be considered for routine use during SVG grafting. Studies with 10-year angiographic patency will be crucial in answering the role of these devices in routine practice.

### 4.4 Radial artery

Vascular diseases of the upper extremities, previous arm trauma or surgery and potential need for the radial artery (RA) for upper extremity arterio-venous fistula are relative contraindications to RA use. Bilateral RA use is infrequent and must be weighted against the potential benefits of using the RA as access for percutaneous interventions. Ulnar compensation should be evaluated using an objective testing, rather than the clinical Allen test. Echo-Doppler, plethysmography and oximetry can all be used; a reduction <50% in the flow in the second digit is generally sign of adequate ulnar compensation (95). The site with the best ulnar compensation should be selected for RA harvesting, independently from arm dominance. RA harvesting can be performed using the open or endoscopic technique. The latter is associated with better cosmetic results and possibly less postoperative arm discomfort, while the data on the effect on RA patency and clinical outcomes are limited (95). While most surgeons agree that intraoperative direct vasodilatation of the artery is important, there is no clear evidence on which vasoactive agent is the most effective:: papaverine, nitrates, calcium antagonists and milrinone have been used in different combinations both locally and systemically (96). There is general agreement that opening of the RA fascia is important to achieve maximal vasodilatation. The RA must be used in situations of limited or no coronary competitive flow. There is not enough evidence to support the use of specific FFR or angiographic cut-offs. A simple rule of thumb is to accept a ratio of 1.3:1 between the RA diameter and the residual lumen diameter of the target vessel, but this has never been formally tested. The RA can be proximally anastomosed to the aorta or to the internal thoracic artery. In the latter situation the RA is more susceptible to failure due to competitive flow and proximal aortic anastomosis should be preferred in case of target vessels with moderate stenosis. The role of postoperative vasodilation is unclear at the moment. Low dose amlodipine can be used for the first postoperative year if tolerated (95).

### 4.5 Endoscopic graft harvesting

The use of endoscopic vein harvesting (EVH) for coronary artery bypass grafting was broadly adapted for its reduction of wound healing complications. Some early studies, the PREVENT IV-Trial (2009) and the ROOBY Trial (2011), suggested the long-term patency and clinical outcomes may be negatively affected (97, 98). However, both studies presented with several limitations.

As a result a randomized controlled trial called the REGROUP-Trial was initiated in 2013. 1,150 patients undergoing CABG at 16 centers of the Veterans Affairs were analyzed (99). The trial showed favorable results for the EVH subgroup with an occurrence of the primary outcome (MAZE: composite outcome of death, myocardial infarction, repeat revascularization) in 13.9% compared to the 15.5% of the open-harvest group (OVH) with a similar pattern that was detected for the individual endpoints. The EVH group also showed a reduced rate (1.4%) of leg-wound infections compared to the OVH (3.1%). Previously reported intermediate results after a median follow-up of 4.7 years of REGROUP confirmed these results (100).

The majority of RCT analyzing both techniques strongly reassured the benefit of EVH. Additionally, these findings are supported by a variety of large meta-analyses, in particular by a review and a consensus statement of the International Society of Minimally Invasive Cardiothoracic Surgery, one of the largest meta-analyses of 76 studies (23 RCT, 53 non-randomized) with a total of 281,459 patients (101). Both, endoscopic and open vein and radial artery harvesting were studied. EVH compared with OVH revealed a significant reduction in wound-related complications, postoperative length of stay, and outpatient wound management resources and increased patient satisfaction. Non-inferiority for EVH was shown based on major adverse cardiac events and angiographic patency. Additionally, the authors emphasize it is reasonable to perform endoscopic radial artery harvesting (EAH) to reduce wound-related complication and to increase patient satisfaction in combination with a reduction in major adverse cardiac events and noninferior patency rate in up to 5 years. Based on their analysis the consensus panel recommended (class I, level B) that EVH and EAH should be the standard of care.

Endoscopic learning curve on patient safety is an essential factor only being compensated by structured learning to obtain high quality medicine. During this period, macroscopic traction, perforation and thermal injuries can be increased during EVH with greater risk of endothelial injury within the graft with an associated risk of early thrombosis. To bridge this gap structured programs should mediate theoretical knowledge, equipment training and a gradual introduction to clinical practical skills with proper patient selection (102). In the REGROUP trial only experienced harvesters were accepted and defined by more than 100 EVH and less than 5% conversion rate to OVH (98). Endoscopic radial artery harvesting should be started and performed from experienced endoscopic vein harvesters to reduce stress of the operator and keep limb-ischemia low.

## 5 Specific topics related to graft treatment

### 5.1 Papaverin good or bad, pharmacological enhancers

Arterial grafts have proclivity to perioperative spasm (0.43%)—Right gastroepiploic and radial artery (RA) more than Internal Mammary Artery (IMA) (103). Mechanistic pathways may include endothelial dysfunction, intracellular calcium dysregulation, mechanical stimuli, drugs, hypothermia, endo or exogenous catecholamines, abrupt cessation of vasodilators, pre-operative nicotine use, release of Endothelin I, Angiotensin II, nor-epinephrine, serotonin, Prostaglandin *F*_2_*α* and Thromboxane *A*_2_ due to cardiopulmonary bypass and surgical stress (104). Not only the spasm makes the surgery technically demanding, but also the perioperative myocardial ischaemia can have suboptimal early and long term outcomes (105). Numerous agents have been used to address arterial spasm. Papaverine, an opiate alkaloid, acts through inhibition of phosphodiesterases and calcium channels. Though safe, Papaverine may depress conduction and prolong the refractory period of myocardial cells and in large doses may produce arrhythmias, tachycardia, increased depth of respiration and slight hypertension, besides skin rashes and gastrointestinal symptoms. Nitroglycerine (NTG) and Sodium Nitroprusside (SNP) are potent Nitrovasodilators and are more potent in treating than preventing vasospasm^4^. Calcium (Ca^++^) antagonists reduce Ca^++^ influx by blocking voltage operated (L-type) Ca^++^ channels, and amlodipine and Cilnidipine also stimulate nitric oxide (NO) release from endothelium (106). Even Botulinum Toxin A, Beta-blockers, Potassium channel opener—Nicorandil, Ca^++^ sensitiser—Levosimendan, Prostacyclin PGI_2_ analogue—Iloprost, Calmodulin inhibitor—Chlorpromazine, guanylyl cyclase activator—Carperitide (Atrial natriuretic peptide) and Fasudil, a Rho-kinase inhibitor have been used to prevent arterial spasm with salutary results in observational studies. Vasodilators may be used topically (spray, wrap, organ bath), intra-luminally or systemically and either as standalone drug, or preferably, as a cocktail to target multiple mechanisms of spasm (105, 106). Because of short duration of action of Papaverine (half life—100 min) and NTG, they can be combined with longer acting Verapamil or Phenoxybenzamine, especially to prevent catecholamine mediated vasospasm of RA conduits with the effect lasting up to 48 h (104). IMA is α1-adrenoceptor predominant, but RA has both α1 and α2 adrenoceptors (106). By blocking α-adrenoceptors, Phenoxybenzamine unmasks the D1 receptor mediated vasodilatation of Dopamine, thus improving the safety profile of RA conduits in CABG (104). A caveat, it may contribute to vaso-spasm (through AT_1_ receptors), if non-catecholamine factors for spasm are predominant (104). Another well established combination is of phosphodiesterase inhibitor milrinone and NTG, which have additive synergistic effects (106). Perioperative intravenous Diltiazem infusion may have a higher antispastic action, and thereby stronger protection from postoperative ischaemia, than NTG (107). Diltiazem and Verapamil should be used for 6–12 months for RA grafts.However, Takeuchi et al. found no benefit of perioperative Papaverine use to increase IMA blood flow (base line—37.2 ± 17.0 vs. post-Papaverine 40.2 ± 19.1 ml/min) (108). Moreover, when given intra-luminally, being an acidic solution (pH 4.4–4.8), it can cause endothelial damage. This can be offset partially by using a low concentration of Papaverine (0.5 mg/ml). Even heparinised blood provides some buffering action. Topical Papaverine has shown inconsistent and variable action. Probably intra-thoracic fascia, fat and muscle around the arterial conduit act as physical barrier. Even additional techniques like periadventitial infiltration of Papaverine and Left Stellate Ganglion Block have been used to prevent peri-operative arterial spasm.

### 5.2 Composite grafting

Revascularization using an *in situ* left internal thoracic artery (ITA)-based composite graft constructed with different conduits, such as right ITA, radial artery, right gastroepiploic artery, and saphenous vein, has advantages of avoiding aortic manipulation and allowing efficient conduit utilization. However, there has been a concern that a composite graft may not supply sufficient blood flow to a wider area of myocardium because it only emanates from a single blood source.

With regard to arterial composite grafting, one study compared coronary flow reserve 2 weeks after coronary artery bypass grafting and demonstrated that bilateral ITA Y-composite graft was not as effective as bilateral ITA *in situ* grafts for improving coronary flow reserve (109). However, another study, which was performed in patients who received bilateral ITAs, demonstrated that revascularization with a Y-composite and bilateral in-situ ITA grafts exhibited a similar pattern of myocardial perfusion improvement at 1 year after surgery (110). A meta-analysis found that use of bilateral ITAs as a composite and bilateral *in situ* graft strategies offer similar clinical outcomes, including a composite of all-cause death, nonfatal myocardial infarction, and repeat revascularization (111). Recent studies demonstrated that long-term (>10 years) clinical outcomes of bilateral ITA composite grafting were comparable to those of bilateral ITA *in situ* grafting and also demonstrated that long-term (>10 years) clinical outcomes of other arterial composite grafting using radial or right gastroepiploic artery were comparable to those of bilateral ITA composite grafting (112–114) A recent extended study of the SAVE RITA (SAphenous VEin vs. Right Internal Thoracic Artery as a Y-Composite Graft) trial demonstrated that the vein composite grafts were comparable to the right ITA composite grafts in terms of 10-year conduit occlusion rates and long-term clinical outcomes (115).

In summary, recent studies showed equivalent results between bilateral ITA composite and *in situ* grafts and between bilateral ITA composite and ITA-based arterial or vein composite grafts in terms of long-term patency rates and clinical outcomes.

## 6 Graft preservation techniques in minimally invasive cardiac surgery

Minimally invasive coronary bypass surgery is carried out through small thoracic incisions and in the majority of cases on the beating heart. At present most frequently a LIMA to LAD is placed (116) but expert groups also perform multivessel minimally invasive CABG using both internal mammary arteries and additional vein grafts or radial arteries (117–121). The principles of graft preservation are essentially the same as in open surgery but there are some specifics. Due to the limited and tangential view during harvesting the IMA trough a mini-thoracotomy more attention needs to be paid at avoiding injury to the graft. Also, the limited length of the IMA in this setting may lead to graft tension (122). This needs to be absolutely avoided. The view on the LIMA and RIMA with videoscopic assistance and robotic approaches is significantly enhanced and more length can be gained. But thoracoscopic or robotic harvesting requires a learning curve with potentially increased rates of graft injury (123, 124). Simulation of endoscopic IMA harvesting in dry lab or wetlab models before applying these techniques clinically is therefore highly recommended. In both techniques exact low power settings of the electrocautery at 15 Watts are key for protection of the graft from thermal injury. Lack of tactile feedback in robotic techniques requires adaption of the operator to a different perception of the surgical field and of the tissue. One unique aspect of totally endoscopic IMA harvesting is that the grafts do not leave the thorax but remain in the closed chest environment and are not even exposed to air during this phase. If the CO2 pressure which is applied to increase space has any consequences for graft integrity and -function has not been studied in detail. Clinical patency data do not suggest this (125). Treatment of the internal mammary artery grafts with vasodilators follows general principles and mostly topical papaverine is applied. Use of intraluminal vasodilators such as verapamil and papaverine has been described both for the mini-thoracotomy direct harvesting technique (126) and for robotic, completely endoscopic techniques (127). For the latter an additional endoscopic injection maneuver is necessary which increases complexity an adds time to the procedure. Therefore, some groups simply clip the graft after harvesting and heparinization and let it auto-dilate (121). Vein grafts have been increasingly used in minimally invasive CABG, specifically in the so-called MICS-CABG technique (128). Venous conduits are harvested from the leg in endoscopic or open techniques and are connected to the ascending aorta using a side-biting clamp or automated connectors. Distal anastomoses are performed on the beating heart using specific exposure and stabilization devices. The grafts in MICS CABG are usually stored in various solutions according to local protocols. We strongly suggest to follow the general recommendations given in this consensus paper. The limited access in MICS-CABG probably does not increase the surgical forces that the graft is exposed to but makes length measurement challenging with the occasional graft being under tension. It is therefore recommended to carry out exact measurements of graft length taking into account the length during different filling states of the heart.

## 7 Discussion

Coronary artery bypass grafting (CABG) is and continues to be the preferred revascularization strategy in patients with multivessel coronary artery disease. Graft selection with the aim of long-term graft patency has been shown to influence the outcomes following CABG surgery. From the very start of CABG procedures saphenous vein grafts (SVGs) together with the internal mammary artery have become the standard of care. Due to the known limitation of reduced long-term patency rates with the utilization of SVGs, serious research efforts have been undertaken to circumvent this drawback. As such, the utilization of alternative conduits (e.g., radial artery, right internal mammary artery) has been increasingly propagated with supporting scientific evidence. However, SVGs remain the most frequently used conduit for non-LAD territories in CABG surgery worldwide and a dogmatic shift to an increased utilization of multiarterial grafts in the foreseeable future seems at least to be in doubt.

**Figure 3:**
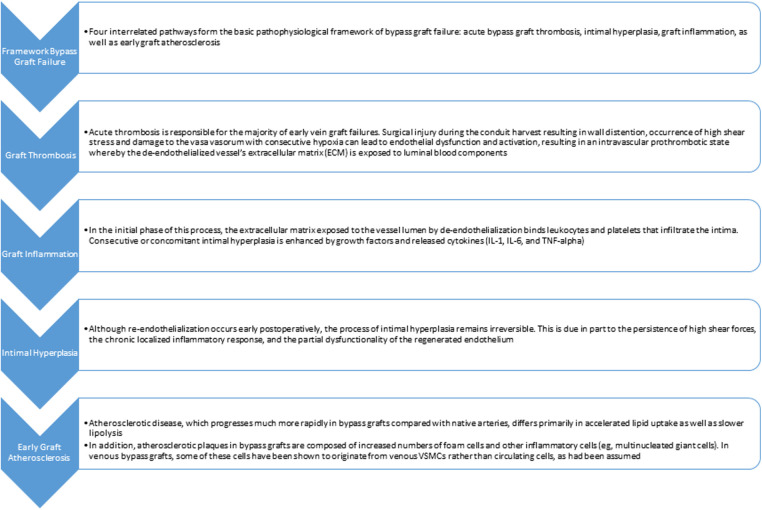
Pathway from bypass graft failure to early graft atherosclerosis.

With an increased understanding of pathophysiological pathways leading eventually to vein graft failure, promising evidence emerged over the last decade in overcoming some of the obstacles and pitfalls of SVG utilization. It seems to be more apparent now that the way how the vein graft conduit is harvested e.g., no touch, with surrounding tissues, treated and stored prior to grafting plays an incremental role in the development of vein graft failure—in the early phases as well as in the long-term (Figure 3).

Although various common practices are well known to be detrimental, a large proportion of cardiac surgeons worldwide—partly due to inadequate awareness—still resist to adopt their practice to avoid deleterious effects to the vein graft.

The increasing utilization of multiarterial grafting techniques with skeleletonized IMAs and radial arteries are legitimate maneuvers to achieve increased long-term graft patency in CABG surgery, however, in light of the broadly used SVGs in the real-world setting, this does not necessarily address the issue at stake.

First, awareness of the harvesting process of SVGs needs to be established. A proper training of the vein harvesting team—usually the least experienced member of the team—needs to focus on the importance of the vein harvesting procedure with the aim to minimize trauma to the vein during harvest.

Next, overdistending the vein conduits with supra-physiological pressures to check for leaks or to increase the lumen of the veins should be abandoned from clinical practice. However, it has to be acknowledged that currently, the utilization of, yet cost-intensive, pressure-controlled syringes in large parts of the world pose an important economical burden (129).

Since the 1980s extensive research and studies challenged the traditional utilization of normal saline or autologous whole blood as storage and flushing solution. Despite a compelling body of evidence that these solutions play an incremental role in the process of vein graft failure, these solutions remain the standard of care in most clinical practices worldwide. At best, a shift to buffered saline that partly alleviates the deleterious effects is observable in recent years.

More recently, research with dedicated solutions (e.g., endothelial damage inhibitors) aiming at the issue of vein graft disease by minimizing the deleterious effects of ischaemia and reperfusion injury on the endothelium provided promising results and further research with long-term data is warranted. External support devices for SVGs significantly reduced the intimal hyperplasia area and thickness, and improved the lumen uniformity, assessed with the Fitzgibbon I classification as recently reported and require further research and attention to improve the outcome in CABG.

In summary the surgical technique is one of the most crucial factors often outruling all other related meassures and efforts. Therefore highest respect has to be paid to the surgical technique and handling of the graft material.
